# Microbials for Agriculture: Why Do They Call Them Biostimulants When They Mean Probiotics?

**DOI:** 10.3390/microorganisms11010153

**Published:** 2023-01-06

**Authors:** Juan Sanjuán, Maria Caridad Nápoles, Daniel Pérez-Mendoza, María J. Lorite, Dulce N. Rodríguez-Navarro

**Affiliations:** 1 Department of Soil and Plant Microbiology, Estación Experimental del Zaidín, CSIC, 18008 Granada, Spain; 2 Departamento de Fisiología y Bioquímica Vegetal, Instituto Nacional de Ciencias Agrícolas (INCA), Carretera San José-Tapaste, Km 3½, Gaveta Postal 1, San José de las Lajas CP 32700, Mayabeque, Cuba; 3 Departamento de Inoculantes, IFAPA Centro Las Torres, Ctra. Sevilla-Cazalla, Km 12,2, Alcalá del Río, 41200 Sevilla, Spain

**Keywords:** inoculants, biostimulants, plant probiotics, plant growth promotion, agriculture sustainability, crop productivity

## Abstract

There is growing interest in using plant-beneficial microorganisms to partially replace chemicals and help reduce the environmental impact of agriculture. Formulated microbial products or inoculants for agriculture contain single strains or a consortium of live microbes, well characterized and biosafe, which can contribute to the growth, health, and development of a plant host. This concept conforms to the definition of probiotics. However, some plant-growth-promoting microorganisms (PGPMs) have been considered a category of biostimulants since some years ago, despite the traditional concept of biostimulants involves substances or materials with no fertilizer value, which in minute amounts promote plant growth. The inclusion of PGPMs together with substances has also involved a significant distortion of the classical concept of biostimulants. Regulations such as the recent EU Fertilizing Products Regulation (EU No. 2019/1009) have incorporated the new definition of biostimulants and included microbials as a subcategory of biostimulants. We discuss that this regulation and the forthcoming European harmonized standards disregard some key features of microbial products, such as the live, true biological nature of their active principles. The factors that determine the complex functional compatibility of plant–microbe associations, and important biosafety issues that concern the intentional release of microbes into the environment, seem to be also ignored. We anticipate that by equating microbials to chemicals, the biological nature of microbial products and their specific requirements will be underestimated, with pernicious consequences for their future development and success.

## 1. Plant-Beneficial Microbes and the Concept of Probiotics

Plants naturally associate with many beneficial microorganisms which have key roles in host development, metabolism, stress adaptation, and health. Plant hosts can selectively attract microorganisms that provide them with a variety of essential functions to adapt their physiology and development to the local environment [1].

The best-known plant-growth-promoting microorganisms (PGPMs) include bacteria and fungi [2,3], but also archaea [4] and viruses [5] can be of benefit to plants. Microorganisms can promote plant growth through a broad range of modes and mechanisms of action. Although beneficial microbes usually display more than one of these mechanisms, they can be grouped according to their principal mode of action and therefore intended biotechnological application.

**Biofertilizers** participate in the provision of nutrients to the plant. Biofertilizers are the broadest and probably best understood category of PGPMs. Nutrient acquisition facilitated by biofertilizers usually involves macroelements such as nitrogen, phosphorus, and potassium, but also microelements such as iron, zinc, copper, etc. [6,7,8]. The mechanisms include atmospheric nitrogen (N_2_) fixation as well as soil nutrient solubilization and mobilization [9].

**Phytostimulators** are rhizosphere or endophyte microorganisms, fungi, and bacteria, which can influence plant growth and development by affecting plant hormone metabolism, either through the production of phytohormones such as auxins, cytokinins, and gibberellins or by interfering with plant endogenous hormone homeostasis [10,11]. This type of PGPM facilitates implantation, accelerates growth, and promotes greater plant vigor, but can also mitigate the damaging effects of abiotic stresses.

**Bioprotectors** are those microorganisms that participate in the induction of plant abiotic stress resistance. Several specific mechanisms have been proposed for microbe-mediated plant tolerance to stresses such as drought or salinity, including volatile compounds, alteration in root morphology, accumulation of osmolytes, exopolysaccharide (EPS) production, and antioxidant defense induction [12,13,14].

**Bioremediators.** These microorganisms participate in the remediation of contaminated soils. Contamination of agricultural soils with heavy metals and other toxic compounds is a significant environmental problem with a strong impact on agriculture and human health. Rhizosphere microbes can significantly affect heavy metal mobility and availability to the growing plant through mechanisms such as the release of chelating agents, acidification and redox changes [15,16].

**Biocontrollers**. Biocontrol Agents (BCAs) are used for suppressing plant diseases and pests. Although less extensively used, biocontrol can also be applied to suppress invasive weeds. Widely used microbial BCAs are certain rhizobacteria and fungi; however. mycoviruses and bacteriophages have the potential to control phytopathogens [5,17]. Effective biocontrollers may compete with pathogens for available nutrients, but often also produce specific compounds (i.e., antibiotics, hydrolytic enzymes, volatiles) that inhibit the growth of the pathogen. Some rhizosphere bacteria and fungi can also contribute to plant immunity through activation of the so-called Induced Systemic Resistance (ISR) in the plant [18,19,20,21].

Beneficial microbes can be isolated from prospective environments and characterized, selected, and used to formulate biotechnological applications, widely known as microbial inoculants. Microbial inoculants may contain pure strains but also complex consortia of plant-beneficial microbes. The application of selected plant-beneficial microorganisms is an important tool to promote crop health and productivity, and an effective alternative that can reduce the use of agrochemicals [22].

Plant microbial inoculants can be defined as commercial products which contain live microorganisms as the active principles, with a specific application to improve health, nutrition or development of a plant host. Microbial inoculants are regulated to include one or more strains of live microorganisms, well-identified and biosafe, with a demonstrated beneficial effect on a well-defined group of target host species (sometimes cultivars) under agronomic conditions. Although adequate formulations of microbial inoculants can determine their application efficacy, the key feature of inoculants is that live microbes are the only essential component. Thus, they fall within the concept of probiotics.

As discussed by the International Scientific Association for Probiotics and Prebiotics (ISAPP), the FAO/WHO-approved definition of probiotics reads, “*Live microorganisms which when administered in adequate amounts confer a health benefit on the host*” [23]. Three major ideas can be threshed from this definition.

First, probiotics mean live, viable microorganisms, either single strains or consortia, which are taxonomically well-identified and of demonstrated biosafety. Dead microbes, microbial products, or microbial components do not fall within the probiotics definition. Excluded are also products with live but undefined microbial contents [23]. The concept of microbial viability included in the definition of probiotics represents the most important and visible difference with other commercial products that also claim beneficial effects but involve a diversity of therapeutical substances or compounds, albeit not live microbes. A well-separated term is prebiotics, which applies to substances selectively utilized by host microorganisms conferring a health benefit [24,25]. Nonviable probiotics are known as paraprobiotics and include so-called inactivated probiotics and postbiotics [26]. Prebiotics and paraprobiotics are also regulated separately from probiotics [25].

Second, the definition of probiotics specifically mentions the importance of adequate administration, particularly dosage, which involves the delivery of efficacious amounts of viable strain(s) but also the frequency of application of the product. Thus, the beneficial effects of probiotics are achieved only within a demonstrated range of live microbial cells, whereas the application of amounts below or above a given threshold level would not ensure the claimed beneficial effects. Probiotics can have different means of administration (i.e., ingestion, skin application), specific target species (in the case of animals), and variable but demonstrated and regulated range of efficacies. The level of efficacy is assumed to fluctuate depending on the status of the host at the time of probiotics application, but also on external variables such as environmental factors and diet. This is an important consideration for probiotic efficacy since live microbes do have their own physiological requirements.

Thirdly, probiotics confer a health benefit on the host, including prevention and/or treatment of diseases and disorders. In humans and animals, health involves avoidance of pathogens/parasites but also balanced nutrition. For instance, vitamin A deficiency can result in blindness, insufficient intake or absorption of Fe results in anemia, whereas incapacity to metabolize lactose results in flatulence and diarrhea. Probiotics may have different mechanisms of action, including direct or indirect antagonism of pathogens, but also nutritional effects such as vitamin synthesis, bile salt metabolism, and production of certain enzymatic activities. Some mechanisms may be widespread and common to many probiotic microbes (i.e., competitive exclusion of pathogens, regulation of intestinal transit, vitamin synthesis, stimulation of the immune system), whereas others can be more specialized such as the production of specific bioactives or direct pathogen antagonism.

Although the term probiotics has been traditionally linked to humans and animals, the FAO/WHO definition does not specify particular groups of host organisms. It was in 2003 that the term probiotics was first used for plant-beneficial microbes, applied to rhizosphere microorganisms capable of reducing diseases caused by pathogens [27], resembling the health-promoting effects of probiotic microbes in the gastrointestinal tract. Since then, several books [28,29,30] and other dedicated publications have used the term probiotics in relation to plant-beneficial microbes [31,32,33,34,35]. Moreover, many microbial products already in the market use the term plant probiotics as a commercial claim.

As for animals and humans, the concept of health applied to plants extends beyond the antagonism of pathogens (i.e., infectious microbes). Plant diseases and disorders can also be produced by abiotic factors such as environmental stresses (i.e., drought, salinity), nutritional deficits, and chemical toxicities [36] A number of nutrient deficiencies and toxicities can result in distinctive types of chlorosis, but also in necrosis, spotting, or flecking. Abiotic disorders can further predispose plants to diseases caused by infectious agents.

In summary, plant-beneficial microbes conform to the concept of probiotics, particularly in their living nature and beneficial effects on hosts, but also in their sites of application and action. Given its microbial richness and its great influence on plant nutrition and health, the rhizosphere resembles the intestinal tract of animals [37], whereas the phyllosphere of plants is clearly like the epidermis of animals.

## 2. Biostimulants

Yakhin et al. [38] have performed an excellent work tracing the history of biostimulants. These authors described the evolution of the often-confusing biostimulant concept. One of the first definitions was offered in the 1990s by R.E. Schmidt as “*Materials of little or no fertilizer value that accelerate plant growth, usually when used at low concentrations*” [39], with a shorter version reading “*materials that, in minute quantities, promote plant growth*” [40]. With slight variations, this concept has prevailed until recently. Thus, biostimulants have been historically considered bioactive substances or compounds, often but not always of complex and undefined composition, which can stimulate plant growth when applied in small quantities [38,41,42,43,44,45,46]. Diverse substances or complex preparations have been categorized as plant biostimulants, including seaweed and plant extracts; humic substances; complex organic materials (from a wide range of sources); biopolymers (i.e., chitosan, alginate) and oligomers; protein hydrolysates and mixtures of peptides and free aminoacids; single aminoacids (i.e., β-aminobutyric acid); inorganic salts (i.e., phosphites); or chemical elements (i.e., silicon). Even pharmaceuticals such as omeprazole, a benzimidazole proton pump inhibitor in mammals, have been reported to increase nitrogen use efficiency in corn and to enhance growth and tolerance to salt stress in tomatoes [47,48,49], although it is unclear that plants possess the known metabolic targets of omeprazole. We are not discussing the reported effects or efficacy of these products; however, it is a common feature that their mode of action is usually unknown, maybe a consequence of their chemical complexity but also of a lack of fundamental studies [38]. Sometimes a combination of various modes of action can be presumed. For instance, it is difficult to separate the positive effects of seaweed and plant extracts owing to their phytohormone contents from those due to their known activities against pathogens (including induction of plant resistance), or to their nutrient contents [50]. Likewise, it is difficult to establish how much of the organic nitrogen in protein hydrolysates and peptides/aminoacid mixtures functions as fertilizer and how much serves as metabolic or physiological activators. For instance peptones, protein hydrolysates commercialized as biostimulants that contain high amounts of N, Fe, and other important nutrients, have significant applications of up to 20 kg ha^−1^ [51].

There is a tendency to tag or market plant biostimulants as natural products, just because of their organic nature or the biological origin of source raw materials of many (but not all) of them. This is the case of seaweed extracts or protein hydrolysates, among others. It is true that seaweeds have been used as organic manures, soil conditioners and fertilizers since ancient times, and as such could be tagged as a natural products. However, whole seaweeds have been mostly replaced by seaweed extracts, which can doubtfully be considered natural products, since they are obtained after various and relatively complex industrial processes [50,52]. Another example are peptones and other protein hydrolysates, which can also be of diverse origins (plants, fishes, ruminants) but are obtained after ever more sophisticated industrial processes. Likewise, chitosan can be obtained from animals (i.e., shrimps, crabs) or fungal sources after significant industrial processing.

Particular confusion in the concept of biostimulant has arisen since 2012, when the consortium of European Biostimulant Industries (EBIC) promoted a re-definition of biostimulants reading “*substance(s) and/or microorganisms whose function when applied to plants or the rhizosphere is to stimulate natural processes to benefit nutrient uptake, nutrient efficiency, tolerance to abiotic stress, and/or crop quality, independently of its nutrient content*” (EBIC, http://www.biostimulants.eu/; accessed on 24 November 2022; [53]). Since then, various authors have supported this industry proposal [6,54,55,56], and with virtually no variations it has been included in the recent European fertilizers regulation approved in 2019 (see below). This new definition of biostimulants represents a significant twist, as it neglects the most important features included in the classical concept of biostimulants. Thus, the positive effects of biostimulants on plant growth have been replaced by an “increase in nutrient uptake, nutrient efficiency or tolerance to abiotic stress”, features that usually, but do not necessarily involve better plant growth or enhanced crop production. Indeed, those newly claimed biostimulant effects seem to be more physiological than agronomical parameters. In addition, the traditional qualification that biostimulants do function at low concentrations or minute amounts has also disappeared from this re-definition. Instead of a positive action “when applied in minute amounts”, there is the understanding that “biostimulants exert their action regardless of their nutrient contents” [54]. However, many commercial products marketed as biostimulants contain significant amounts of macro and micronutrients and, as discussed above, it is often difficult to discern nutritional effects from other modes of action.

Last but not least, another important modification in the biostimulant concept is the significant enlargement of the nature of biostimulants from chemicals (substances/compounds) to include also microorganisms. In a report on biostimulants du Jardin [43] stated that plant-beneficial microorganisms were not considered biostimulants by the research community. Two years later, in another report also co-authored by du Jardin, the new concept of biostimulants was proposed to include microorganisms as biostimulants [57].

This notable change in the concept of biostimulants has been argued around two ideas: (1) that biostimulants are intrinsically diverse, and (2) that the important feature is what they do and not what they are. With these arguments, du Jardin [54] explains that “*any definition of biostimulants should focus on their agricultural functions and not on the nature of their constituents nor their modes of action*”. However, this neglects the true biological nature of living microorganisms. Compared to chemicals, biologicals exhibit specific features that are critical not only for their industrial production, but also to determine their efficacy range, biosafety, and ecological impact, which must be specifically considered in their regulations. Live microorganisms are biological entities that have their own capacities to respond, adapt, evolve, and actively interact with the environment and each one of its components. As discussed by Yahkin [38], it is questionable that living microbial products can be referred to as biostimulants. In no case should live microbes be equated to substances.

## 3. The New EU Regulation on Biostimulants

The previous European regulation on fertilizers (EC No. 2003/2003) has been repealed by the recently approved EU Fertilizing Products Regulation [Regulation (EU) No. 2019/1009]. In short, the classical concept of fertilizer (material that provides nutrients for plants) has been replaced by “fertilizing products”, which include materials, substances, microorganisms, and mixtures intended to provide plants with nutrients or improve their nutrition efficiency. The new regulation introduces seven product function categories (PFCs), one of which is plant biostimulants.

As discussed above, significant confusion has been generated after the change in the biostimulant concept and definition promoted by the EBIC. With few changes, this EBIC definition has been transposed into the new Regulation (EU) No. 2019/1009, which entered into force in July 2022. This regulation defines plant biostimulants as “*a fertilising product the function of which is to stimulate plant nutrition processes independently of the product’s nutrient content with the sole aim of improving one or more of the following characteristics of the plant or the plant rhizosphere: nutrient use efficiency, tolerance to abiotic stress, quality traits or increasing the availability of confined nutrients in the soil or rhizosphere*”. Plant biostimulants are included in product functional category 6 (PFC6), which distinguishes between microbial (PFC6A) and non-microbial biostimulants (PFC6B) and indicates that a microbial plant biostimulant shall consist of a microorganism or a consortium of microorganisms referred to in CMC 7: Microorganisms. CMC 7 (Component material category 7) further details that “*An EU fertilising product belonging to PFC 6(A) may contain microorganisms, including dead or empty-cell micro-organisms and non-harmful residual elements of the media on which they were produced*”. Together with this wide concept of microorganisms, paradoxically the list of microorganisms accepted as microbial biostimulants is limited to *Azotobacter* spp., Mycorrhizal fungi, *Rhizobium* spp., and *Azospirillum* spp. By listing this reduced number of microorganisms, this regulation indirectly excludes many well-known PGPMs such as *Bacillus*, *Pseudomonas*, *Trichoderma*, and many others. Moreover, Regulation (EU) No. 2019/1009 specifically excludes microorganisms that can be used as biocontrollers, which remain under Plant Protection Products Regulation (EU) No. 1107/2009. Why are so many PGPMs excluded? Why are microbial biocontrollers regulated separately from other PGPMs?

Microbial products for agriculture are based on essentially the same active principles (live microbes = probiotics), which are produced by similar industrial processes (fermentation, formulation), are managed and applied also similarly, with a similar purpose: to achieve a beneficial effect for a given crop/host. Then, why are certain microbials regulated separately based on their mechanisms of action? Moreover, why are so-called microbial biostimulants regulated together with chemicals (organics and inorganics)?, which are of a totally different nature and modes of action from microbials.

It appears that when it comes to regulating products based on plant-beneficial microorganisms, it seems equally important what they are, what they do, and how they do it. This appears contrary to the arguments of du Jardin [54] that, regarding biostimulants, the important thing is what they do and not what they are.

We forecast that by equating microbials and chemicals, the biological nature of microbial products will be underestimated, with pernicious consequences for their development and their acceptance by farmers, and therefore for their success.

The disparagement of the biological nature of microbes can be already presumed from the EU 2019/1009 definition of microbial biostimulant, which states that “*it may contain microorganisms, including dead or empty-cell micro-organisms*”. Since a product based on dead microbes is likely closer to an organic mixture than to a true microbial product, should such a product still be tagged as microbial biostimulant?

One of the key features of microbials is the requirement for optimal numbers of viable and active microbial units (VAMUs; being either single cells, spores, mycelial fragments, etc.), which ensure the efficacy of the product. The optimal VAMUs can be very variable depending on the class of microorganisms and mechanism of action, but also are very dependent on specific features of the target host (e.g., seed size) and the form of application (on soil, seeds, leaves). VAMUs usually involve critical minimum numbers, but also a maximum range of viable active units above which detrimental or non-desired effects could be induced. Contrary to chemicals, VAMUs must be guaranteed not just at the time of production, but also during the whole shelf-life period until product acquisition by the user. Regulations of microbial inoculants worldwide (USA, Mercosur, India, etc.; reviewed in [58]), lay particular emphasis on these features, viability and activity, to define minimum quality standards (see for instance, [59]). Can regulations determine the quality of microbial products if these can contain dead or live microbes?

Additional symptoms of underestimation of microbial key features appear in the publication of Ricci and collaborators [53], who listed a number of guiding principles to be incorporated in the forthcoming harmonized European standards. These guidelines developed by EBIC are meant to facilitate the implementation of the European biostimulants regulation. In our opinion, these guidelines appear to be general recommendations to justify any claimed effects of biostimulants, rather than rigorous requirements to ensure their efficacy.

Regarding efficacy dosage, Ricci et al. [53] indicate that since it may be difficult to estimate minimum effective doses of microbial biostimulants, then field-generated data may not be necessary, and the recommended dose could be justified, for instance, with data from preliminary studies so that the lack of precise or conventional dose justification data should not preclude the placing of the biostimulant on the market. Minimum quality standards in other countries require defined VAMUs at all times, from production throughout the shelf life of the microbial products. Very often, companies not only comply with those standards, but their products are well above the official requirements (i.e., [59]). Regulations that do not guarantee such minimum standards usually lead to low performance and consequently abandonment of microbial products [59].

Also regarding EBIC recommendations, it is particularly worrying to read that “*in the case of products that improve the availability of nutrients (notably micro-organisms), soil types and soil conditions can be more relevant than crop type itself when designing trials*” [53]. Certainly, the edapho-climatic conditions are key variables for field trials, and these variables make the principal difference between laboratory and field experiments. Not by chance, the great majority of microbes that appear promising in laboratory experiments and are published in papers, do not perform in field tests. Nevertheless, performance with a particular host species and genotype is *conditio sine qua non* to be considered promising and therefore to go to field trials, which should be conducted with the same host species and genotype for which laboratory data have been acquired. Performance in a given crop species should not be extrapolated from other related or unrelated species. To be rigorous, extrapolation should not be done even for cultivars of the same species. Instead, Ricci and collaborators [53] proposed crop groupings to justify biostimulant claims, suggesting that data obtained in a given crop can justify similar claims in others. Again, this neglects the particularly strict and often complex compatibility rules that govern the microbe–plant interactions. Even micorrhizal fungi, which display broad host ranges and can infect most land plants, do show significantly wide functional compatibility ranges, from great plant growth improvements to host depression, and these compatibility ranges are mostly dependent on both fungal and host plant genotypes (i.e., [60]). Likewise, *Azospirillum* inoculant performance in cereals is very dependent on the plant genotype, which determines key features such as the initial root colonization and the later survival of the bacterium [61,62]. It is even more important to consider this functional compatibility in microbe-plant interactions which are known to be governed by severe genotype–genotype specificity, for instance the Rhizobia-legume symbioses [63,64].

Besides the importance of plant and microbial genetic backgrounds, functional compatibility is further modulated by other factors such as crop management practices and edapho-climatic conditions. Thus, when it comes to microbials, all factors including edaphic, climatic, operational, physiological, and especially genetics, are important for the efficacy of the product, and therefore all must be considered when designing field trials and justifying product claims.

Other important quality criteria are the identification of microbes at the species and strain levels, as well as the host species and cultivar(s) of application. The EU 2019/1009 regulation has provided a positive list of microroganisms accepted for microbial biostimulants. As mentioned above, this positive list is exclusionary of many well-known beneficial microbes. There have been already claims for its enlargement and clarification [65,66]. We agree that this list should be as large and comprehensive as possible. However, taxonomic criteria should go beyond the genus or species level, up to the strain level. As discussed previously, the genotype-genotype interaction usually determines the functional compatibility and the outcome of the plant-microbe associations. In countries such as Brazil or Uruguay, microbial products can only be elaborated with well-characterized strains of microorganisms, which are approved and recommended by the authorities after intensive (public and/or private) research and field testing. Usually, this involves interesting public–private research collaborations and ensures that the best-performing microbial strains are applied to the crop varieties commonly grown in particular geo-climatic areas. At the same time, local authorities do provide the recommended strains to all the authorized inoculant industries (as long as they comply with intellectual property rights). This encourages competition among companies through the quality of their formulations, while the use of well-demonstrated high-performance microbials is ensured.

Another important issue that specifically concerns microbials is biosafety. Some authors have warned that the taxonomic criteria alone do not guarantee biosafety, since taxonomy does not consider particular features of individual strains [67]. As indicated by Barros-Rodríguez et al., [68], the release of microorganisms must be supervised to prevent the incorporation of pathogens. These authors have proposed that specific bioassays and biosafety criteria should be followed to predict the impact of released microbials on humans and the environment.

## 4. Conclusions

Biotechnological products based on PGPMs can enhance crop productivity and contribute to the sustainability of agriculture. Since they are based on well-defined live microorganisms and provide a benefit to a host, they conform to the concept of probiotics. The inclusion of PGPMs together with substances as biostimulants has involved not only a significant distortion of the classical concept of biostimulants, but has also led to the implementation of regulations that underestimate key features of microbials. The true biological nature of plant probiotics and the complexity of interactions with their hosts and the environment should be specifically considered and enforced in regulations, to ensure their successful development and adoption by end users.

## Data Availability

No new data were created in this study. Data sharing is not applicable to this article.
